# The Impact of Cannabidiol Treatment on Anxiety Disorders: A Systematic Review of Randomized Controlled Clinical Trials

**DOI:** 10.3390/life14111373

**Published:** 2024-10-25

**Authors:** Carly de Faria Coelho, Rodolfo P. Vieira, Osvaldo Soares Araújo-Junior, Pedro Sardinha Leonardo Lopes-Martins, Larissa Gomes dos Santos, Lucas Danilo Dias, Alberto Souza de Sá Filho, Patrícia Sardinha Leonardo, Sandro Dutra e Silva, Rodrigo Alvaro Brandão Lopes-Martins

**Affiliations:** 1 Laboratory of Biophotonics and Experimental Therapeutics—LABITEX, Universidade Evangélica de Goiás—Unievangélica, Av. Universitária Km 3.5, Anápolis 75083-515, GO, Brazil; carlyfcoelho@gmail.com (C.d.F.C.); larissagomessantos2003@gmail.com (L.G.d.S.); 2 Laboratory of Exercise Immunology, Universidade Evangélica de Goiás—Unievangélica, Av. Universitária Km 3.5, Anápolis 75083-515, GO, Brazil; rodrelena@yahoo.com.br; 3 Laboratory of Applied Neurosciences, Universidade Evangélica de Goiás—Unievangélica, Av. Universitária Km 3.5, Anápolis 75083-515, GO, Brazil; psi.osvaldo@gmail.com (O.S.A.-J.); pdlopesmartins@gmail.com (P.S.L.L.-M.); alberto.filho@unievangelica.edu.br (A.S.d.S.F.); 4 Laboratory of New Materials, Post-Graduate Program in Pharmaceutical Sciences, Pharmacology and Therapeutics, Universidade Evangélica de Goiás—Unievangélica, Av. Universitária Km 3.5, Anápolis 75083-515, GO, Brazil; lucas.dias@docente.unievangelica.edu.br; 5 Laboratory of Health Technologies—LATES, Universidade Evangélica de Goiás—Unievangélica, Av. Universitária Km 3.5, Anápolis 75083-515, GO, Brazil; pssardinha@yahoo.com.br; 6 Laboratory of Natural History of Cerrado, Post-Graduate Program in Pharmaceutical Sciences, Pharmacology and Therapeutics, Universidade Evangélica de Goiás—Unievangélica, Av. Universitária Km 3.5, Anápolis 75083-515, GO, Brazil; sandrodutra@unievangelica.edu.br; 7 Post-Graduate Program in Bioengineering, Universidade Brasil, Av. Carolina Fonseca, Itaquera, São Paulo 08230-030, SP, Brazil

**Keywords:** cannabidiol, anxiety, psychiatry, neuropsychological effects, RCT

## Abstract

Generalized Anxiety Disorder (GAD) is a common psychiatric condition characterized by persistent and excessive worry, often accompanied by dysautonomic symptoms that significantly impact patients’ well-being. Cannabidiol (CBD), a non-psychoactive compound derived from cannabis, has shown potential as an anxiolytic through its partial agonism of the 5HT-1A receptor and its negative allosteric modulation of CB1 receptors, which may help mitigate the anxiogenic effects of tetrahydrocannabinol (THC). This study evaluates the impact of CBD on individuals diagnosed with various anxiety disorders, comparing its effects to placebo and conventional pharmaceutical treatments through a systematic review of randomized controlled trials (RCTs). A systematic search of RCTs published between 2013 and 2023 was conducted across three databases using the terms “cannabidiol” and “anxiety”. Out of the 284 articles identified, 11 met the eligibility criteria. The studies reviewed varied widely in terms of the types of anxiety disorders and CBD dosages examined, leading to results that were often contradictory. Despite these conflicting outcomes, the data suggest that CBD may reduce anxiety with minimal adverse effects when compared to a placebo. However, further RCTs with improved methodologies, encompassing a broad range of doses and continuous CBD administration across specific anxiety disorders, are needed. Unlike previous studies and meta-analyses, this review encompasses a broader spectrum of anxiety disorders and a variety of study designs and dosages, providing a more nuanced understanding of CBD’s potential efficacy across different conditions.

## 1. Introduction

*Cannabis sativa*, commonly known as marijuana or hemp, is an indigenous plant originating from East Asia that synthesizes numerous chemical compounds. Currently, 554 chemicals have been identified, including 113 phytocannabinoids and 120 terpenes, which contribute to its distinct aroma [1,2,3]. Marijuana, the most consumed illicit drug in the United States in 2019, has a centuries-long history of recreational use. Beyond its recreational appeal, it exhibits diverse medicinal properties such as analgesia, anti-inflammation, immunosuppression, anticonvulsant effects, and antiemetic properties. However, its use is not without adverse consequences, including documented impacts on cognition, cardiovascular function, and other physiological systems [3].

∆9-tetrahydrocannabinol (∆9-THC) is the principal psychoactive compound in cannabis, while cannabidiol (CBD) is its main non-psychoactive counterpart [2,4]. CBD, first isolated from Mexican marijuana in the late 1930s, gained structural elucidation in 1963 by Mechoulam and Shvo after extraction from purified cannabis preparations [5,6,7]. In the United States, the Food and Drug Administration (FDA) has sanctioned the use of highly concentrated herbal CBD preparations for treating seizures associated with Dravet and Lennox–Gastaut syndromes and tuberous sclerosis complex [8]. CBD can also be synthetically produced, yielding a pure form [9,10]. The comparative efficacy of natural versus synthetic CBD shows similarities in pharmacokinetics and effects, with natural formulations potentially containing other phytocannabinoids that may influence their effects [9].

While significant research has focused on the chemistry and pharmacology of cannabinoids, the clinical efficacy of CBD in treating specific anxiety disorders still requires robust investigation, especially given its approved use in some conditions. This gap in the literature highlights the need for comprehensive evaluations that explore not only the potential benefits of CBD across a spectrum of anxiety disorders but also the variations in effectiveness based on dosage, formulation, and specific patient populations.

THC, a prominent phytocannabinoid, interacts primarily with the CB1 receptor, and its effects on anxiety are dose-dependent, showing both anxiogenic and anxiolytic responses. In contrast, CBD is believed to exhibit anxiolytic properties through partial interaction with the 5HT-1A receptor, among other mechanisms, making it a compound of growing interest for treating anxiety disorders.

Generalized Anxiety Disorder (GAD) is a prominent psychiatric morbidity characterized by persistent and excessive worry, potentially accompanied by dysautonomic physical symptoms, significantly impacting patients’ quality of life. Although its pathophysiology remains uncertain, a multifactorial etiology is evident, involving environmental, psychosocial, and possibly genetic factors. Neurotransmitter dysregulation, notably reduced serotonergic activity and increased noradrenergic activity, contributes to GAD [5,8]. The current therapeutic options for GAD include benzodiazepines, azapirones, and antidepressants. Benzodiazepines, for instance, work by enhancing GABA activation, which helps alleviate somatic symptoms of anxiety. However, they pose risks of abuse, dependence, and withdrawal, particularly when not properly tapered.

This study aims to fill the gaps in the existing literature by systematically evaluating the effects of CBD on individuals diagnosed with various types of anxiety disorders, including Generalized Anxiety Disorder (GAD), Social Anxiety Disorder (SAD), and others [11,12], through a comprehensive review of randomized controlled trials (RCTs). By examining a broader spectrum of anxiety-related conditions and considering the diversity in study designs and CBD dosages, this review seeks to provide nuanced insights that can inform both clinical practice and future research.

## 2. Methodology

### 2.1. Study Design

Numerous studies have explored the effects of CBD on anxiety arising from various causes. Our primary inquiry is focused on determining whether CBD exhibits an anxiolytic effect. If an affirmative response is obtained, we aim to ascertain whether CBD’s effects surpass those of conventional pharmaceutical treatments. To address these questions, we opted for a systematic review of studies investigating anxiety treatment with CBD, covering a span of 11 years.

### 2.2. Search Strategy

We conducted searches in three databases: Medline from PubMed and Virtual Health Library (VHL), Central from Cochrane Library, and Lilacs from VHL. Central compiled studies from Embase, PubMed, the International Clinical Trials Registry Platform (ICTRP) of the World Health Organization, and Clinical Trials from the National Library of Medicine (CT.gov).

The PICOS (population, intervention, comparison/control, outcome, and study design) acronym guided our search strategy and the establishment of inclusion/exclusion criteria [13]. For this review, P represents people diagnosed with anxiety; I represent cannabidiol; C represents placebo or other conventional pharmaceutical treatment; O represents effects on anxiety; and S represents randomized controlled trials (RCTs).

Keywords and MeSH terms were combined using Boolean operators as follows: (“cannabidiol” AND “anxiety”). Filters were applied for the publication time range, study design, and languages to refine the search strategy. To ensure systematicity and guide study selection, we adhered to the Preferred Reporting Items for Systematic Reviews and Meta-Analysis (PRISMA) guidelines [14].

### 2.3. Selection Criteria

#### 2.3.1. Eligibility Criteria

Inclusion criteria for this systematic review encompassed only RCTs presenting results on the effects of CBD on anxiety, studies published between 2013 and 2023, and studies written in English, Spanish, or Portuguese.

#### 2.3.2. Exclusion Criteria

Excluded from this review were RCT protocols, studies reporting results on components other than cannabidiol in cannabis, studies with only abstract publications, studies preceding January 2013, and studies in languages other than those specified in the inclusion criteria.

### 2.4. Study Identification

The inclusion of studies was determined by consensus between two investigators, with the final selection reviewed by a third experienced researcher.

Phase 2—The same four investigators (OS, LG, PSLM, and ALR) independently screened the articles included in phase 1 by reading them in full, adhering to predetermined eligibility criteria. The inclusion of studies was determined by consensus, with the final selection reviewed by a third experienced researcher as in phase 1.

Phase 3—The same four investigators from phase 2 independently assessed the risk of bias in the RCTs included in phase 2 using the Cochrane Collaboration’s Tool [15].

### 2.5. Data Extraction 

Data extraction included author(s), year of publication, sample size, patient characteristics (sex, age range, the type of anxiety, and the use of concomitant anxiolytic therapy), CBD dose range, anxiety measurement methods, effects on anxiety, comparisons with conventional treatments or placebos, side effects, and study limitations.

## 3. Results

### 3.1. Search Output and Flow

**Figure 1:**
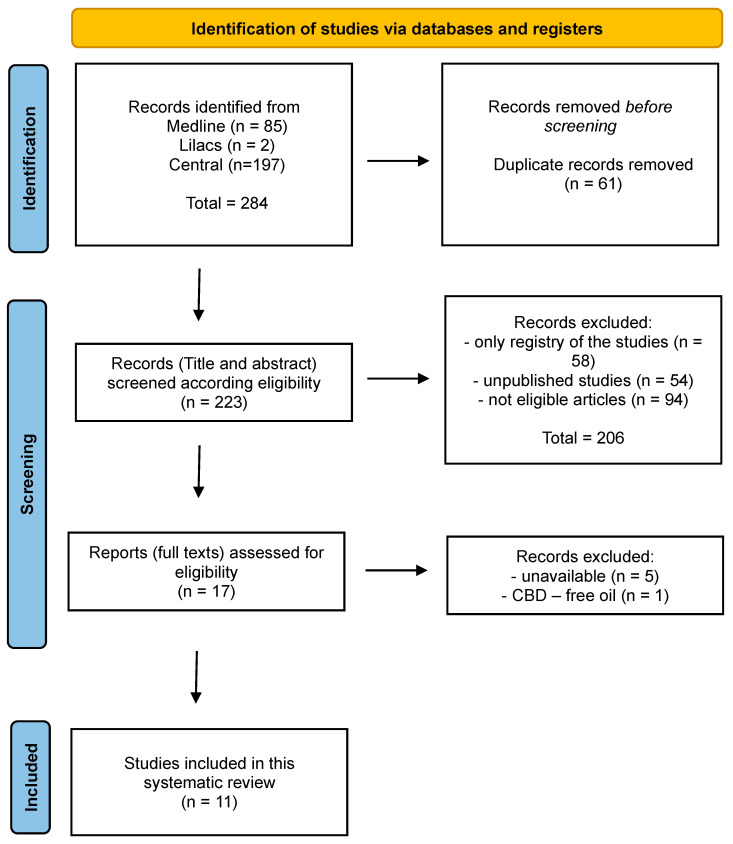
Overview of the screening and selection process for the systematic review according to PRISMA.

As illustrated in Figure 1, our initial search identified 284 articles: 85 from Medline, 197 from Central, and 2 from Lilacs. After removing duplicates, 223 articles remained for screening based on the eligibility criteria by titles and abstracts. A total of 207 records were excluded during this phase, leaving 16 articles selected for a comprehensive assessment.

Upon an in-depth examination of the 16 records, 1 article was excluded as it did not utilize CBD in the treatment of anxiety. Consequently, 15 records were included in this systematic review.

### 3.2. Outcomes

As outlined in Table 1, a total of 673 individuals were assessed to evaluate the effects of CBD on anxiety induced by various factors. While most studies included both sexes, Linares et al. (2019) [16] and Meneses-Gaya et al. (2021) [17] exclusively enrolled men in their samples, and Kwee et al. (2022) [18] focused solely on women.

The selected individuals across studies were generally at least 18 years old, and anxiety induction methods varied, with public speech being the predominant trigger. Other causes of anxiety in the reviewed studies included abstinence from illicit drugs, paranoid thoughts, post-traumatic stress disorder, and testing situations (refer to Table 1).

Notably, three studies indicated that participants were undergoing concomitant anxiolytic/antidepressant therapy (refer to Table 1). Meneses-Gaya et al. (2021) [17] reported hospitalized volunteers receiving psychotherapy and benzodiazepines as needed. Hutten et al. (2022) [19] administered both THC and CBD concurrently in one group. In the study by Mongeau-Pérusse et al. (2022) [20], volunteers were using diphenhydramine and/or trazodone for insomnia, with the stipulation that these medications were not taken within 24 h before the cue-induced craving session.

**Table 1:** Demographic and clinical characteristics of anxiety.

Author(s) and Year of Publication	Sample Size	Sex	Age Range	Cause of Anxiety	Concomitant Anxiolytic Therapy
Zuardi et al., 2017 [21]	60	M/W	18–35	Public speech	_____
Hundal et al., 2018 [22]	32	M/W	18–50	Paranoid thoughts	_____
Linares et al., 2019 [16]	57	M	____	Public speech	_____
Hurd et al., 2019 [23]	42	M/W	21–65	Abstinence of heroin use	_____
Appiah-Kusi et al., 2020 [24]	58	M/W	____	Social stress—speech	_____
Faria et al., 2020 [25]	24	M/W	____	Public speech of volunteers with Parkinson’s disease	_____
Meneses-Gaya et al., 2021 [17]	31	M	18+	Abstinence of crack cocaine use	Psychotherapy and benzodiazepine
Bloomfield et al., 2022 [26]	24	M/W	18–70	Stress	_____
Hutten et al., 2022 [19]	26	M/W	_____	Abstinence of illicit drugs and alcohol	1 group received THC and CBD
Bolsoni et al., 2022 a [27]	33	M/W	18–60	PTSD	_____
Bolsoni et al., 2022 b [28]	33	M/W	18–60	PTSD	_____
Kwee et al., 2022 [18]	80	W	18–65	Social anxiety	_____
Mongeau-Pérusse et al., 2022 [20]	78	M/W	18–65	Cocaine use	Diphenhydramine and/or trazodone was provided for insomnia but not within 24 h before the cue-induced craving session
Stanley et al., 2022 [29]	32	M/W	18–55	Test anxiety	______
Gournay et al., 2023 [30]	63	M/W	18–55	High trait worriers	_____

Note: M = men; W = women; PTSD = post-traumatic stress distress; THC = delta-9-tetrahydrocannabinol; CBD = cannabidiol.

The CBD doses in the study protocols varied from 13.75 mg to 800 mg, with all protocols incorporating a placebo group. Notably, only one study [21] included a conventional pharmaceutical treatment group for comparison with CBD groups and the placebo (refer to Table 2).

Upon an analysis of the 15 studies included in this systematic review, it is observed that nine studies (60%) reported that a single dose of CBD, administered in different amounts, did not exhibit a beneficial effect on anxiety induced by various causes. For instance, 300 mg of CBD failed to reduce anxiety triggered by traumas [27,28], crack cocaine abstinence [17], agoraphobia [18], high trait worriers [30], or test-induced anxiety [29]. Similarly, higher doses of CBD (600 mg), administered as a single dose, also showed no reduction in anxiety, as demonstrated by Hundal et al. (2018) [22], Appiah-Kusi et al. (2020) [24], Bloomfield et al. (2022) [26], and Stanley et al. (2022) [29]. Intriguingly, in two of these studies [22,29], the administration of 600 mg of CBD actually increased anxiety symptoms. A repetitive administration of CBD (800 mg), given daily for 12 weeks, also failed to reduce anxiety in individuals with cocaine use disorder [20].

Interestingly, in studies that reported beneficial effects of CBD on anxiety, the doses varied from 300 mg to 800 mg, and the triggers for anxiety were diverse (refer to Table 2). For instance, in the study by Hurd et al. (2019) [23], both 400 mg and 800 mg of CBD in a single dose demonstrated anxiety reduction in individuals experiencing heroin abstinence. A single dose of 300 mg of CBD proved effective in alleviating anxiety induced by public speech, as indicated in the studies by Zuardi et al. (2017) [21], Linares et al. (2019) [16], and Faria et al. (2020) [25]. Conversely, the study by Gournay et al. (2023) [22] revealed that the beneficial effects on anxiety with 300 mg of CBD only manifested with a long-term administration of the compound, specifically with daily doses over 2 weeks.

While CBD, administered alone, did not exhibit any anxiolytic effect in the studies reviewed, Hutten et al. (2022) [19] reported that CBD completely relieved anxiety induced by THC when the baseline anxiety was low and partially alleviated anxiety when the baseline anxiety was moderate. In another study by Bolsoni et al. (2022a) [27], cognitive impairment measures related to trauma recall were significantly lower in patients who received CBD compared to the placebo (refer to Table 2). Furthermore, CBD demonstrated a beneficial effect in alleviating anxiety induced by a nonsexual trauma; however, this effect was not observed in the context of a sexual trauma (refer to Table 2).

As indicated in Table 2, the Visual Analog Mood Scale (VAMS) emerged as the most frequently employed tool to measure anxiety, utilized in 50% of the studies included in this review. Following closely were the Visual Analog Scale for Anxiety (VAS-A; 41.6%), Beck’s Anxiety Inventory (BAI; 41.6%), and State–Trait Anxiety Inventory (STAI; 33.3%).

Several other tools, including the Emotional Stroop Test (EST), Fear Questionnaire (FQ), Liebowitz Social Anxiety Scale (LSAS), Self-Statements during Public Speaking Scale (SPSS), Westside Test Anxiety Scale (WTAS), State-Trait Anxiety Inventory IDATE, an anxiety subscale of the Depression, Anxiety, and Stress Scales-21 (DASS-A), and Somatic-symptom scale-8 (SSS-8), were also employed for similar purposes (refer to Table 2).

Physiological measures of anxiety, such as blood pressure and heart rate, were commonly utilized across studies (refer to Table 2).

Four studies (Hundal et al., 2018; Meneses-Gaya et al., 2021; Kwee et al., 2022; Gournay et al., 2023) [17,18,22,30] reported that the most prevalent adverse effects attributed to CBD were tiredness, drowsiness, dizziness, and nausea (refer to Table 2). Despite the great variability of the study limitations, the ones that were mentioned more often were the small sample size, followed by the administration of a single dose of CBD and the subjectivity of the self-reported tools used to measure anxiety.

### 3.3. Quality Appraisal—Assessment of Risk of Bias

**Figure 2:**
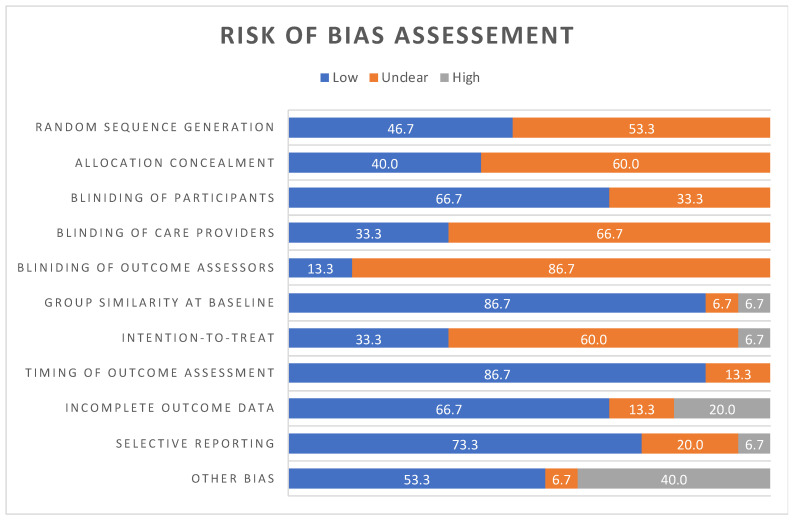
Note: consider numbers as percentages (%).

All 15 randomized controlled trials (RCTs) underwent a risk of bias evaluation using the Cochrane Collaboration’s Tool (15). The assessment revealed that crucial items, including random sequence generation (53.3%), allocation concealment (60%), the blinding of care providers (66.7%), the blinding of outcome assessors (86.7%), and the intention to treat (60%), were categorized as having an unclear risk of bias (refer to Figure 2).

Conversely, 66.7% of all studies included in this review were deemed to have a low risk of bias for both the blinding of the participants and incomplete outcome data. Additionally, 86.7% were classified as having a low risk of bias for both group similarity at baseline and the timing of the outcome assessment. For the selective reporting item, 73.3% of all studies were classified as having a low risk of bias (refer to Figure 2).

The assessment of “other bias” in Figure 2 indicates a relatively balanced classification between a low (53.3%) and high risk of bias (40%).

## 4. Discussion

This review stands out by including a wide range of anxiety disorders and analyzing the impact of different dosages and study designs, offering insights that extend beyond the findings of recent meta-analyses. This discrepancy might be attributed to a notable limitation present in many of the studies analyzed in this review—the administration of suboptimal doses of CBD in a single application. Another plausible explanation lies in the hypothesis that different stressors may engage distinct mechanisms in inducing anxiety, leading CBD to elicit varied responses based on the nature of the stressor. This review stands out by including a wide range of anxiety disorders and analyzing the impact of different dosages and study designs, offering insights that extend beyond the findings of recent meta-analyses.

Studies, such as the one by Guimarães et al. (1990) [31], have demonstrated that CBD generates an inverted U-shaped dose–response curve, particularly identified in preclinical studies, wherein intermediate doses of CBD provide anxiety relief in experimental models. This bell-shaped response curve pattern was also observed in recent clinical trials, including those by Zuardi et al. (2017) [21] and Linares et al. (2019) [16], both included in this review.

In alignment with this dose–response curve pattern, we noted that out of the four studies employing the public speaking model to induce anxiety, three of them [16,21,25], administering a moderate dose of 300 mg of CBD before the public speaking test, reported beneficial effects on anxiety. In contrast, a study by Appiah-Kusi et al. (2020) [24], using 600 mg of CBD, did not yield a similar result.

Conversely, the same 300 mg of CBD, which demonstrated efficacy in relieving anxiety induced by the public speaking test, failed to produce beneficial effects in studies focusing on anxiety induced by other causes, such as sexual trauma, crack cocaine abstinence, or agoraphobia, or anxiety experienced by high trait worriers [17,18,27,28,30]. Similarly, 600 mg of CBD not only failed to reduce anxiety caused by paranoid thoughts or tests among college students but exacerbated the symptoms [22,29].

Contrary to our previous observations, studies focused on anxiety induced by illicit drug abstinence revealed an opposing trend. While higher doses of CBD (400 mg and 800 mg), administered as a single dose, successfully alleviated anxiety induced by heroin abstinence [23], 300 mg of CBD failed to reduce anxiety caused by crack cocaine abstinence [17]. Furthermore, even a lower dose of 13.75 mg of CBD proved ineffective in reducing anxiety caused by illicit drugs in general, including alcohol [19].

In an experimental study with rats, Galaj et al. (2020) [32] demonstrated that CBD could be a potential treatment for cocaine addiction, albeit with limited effectiveness for high doses of cocaine. Translating this to human drug addiction, where it is challenging to control the dose of self-taken illicit drugs, may explain the lack of beneficial effects observed with lower doses of CBD, as illustrated in the examples above, despite varying abstinence periods ranging from 7 days to a month. Building on this perspective, Mongeau-Pérusse and colleagues (2022) [20] explored long-term treatment with a higher dose of CBD (800 mg) for individuals with cocaine use disorder yet failed to find any beneficial effects for this population.

Stanley and colleagues (2022) [29] proposed that CBD might exert specific anxiolytic effects on certain aspects of anxiety, such as cognitive, physiological, or behavioral responses, rather than broadly acting on overall anxiety symptoms. This aligns with our initial thoughts in the discussion and helps explain the inconsistency in results across the studies reviewed.

Adding another layer of complexity, the controversial effects of CBD on anxiety symptoms and the absence of a clear dose–response pattern may be attributed to the sensitivity of the tools used to test anxiety. Some tools may be sensitive to specific classes of medication for anxiety but less responsive to others, including CBD [33].

As highlighted by Kwee et al. (2022b) [34], despite the well-established safety and tolerability profile of CBD, the lack of knowledge concerning the range between the minimum and maximum tolerated doses of this compound, as well as the optimal timing between CBD administration and experimental models of anxiety, presents a significant limitation noted in some studies. This knowledge gap may contribute to the conflicting results observed in this review.

Several studies [17,18,22,30] reported adverse effects such as tiredness, somnolence, nausea, and sedation following CBD administration, aligning with findings from Huestis et al. (2019) [35], which suggested that CBD boasts a favorable side effect profile compared to other drugs.

Another critical aspect for consideration is the variability and subjectivity inherent in the anxiety measurement scales used in the studies included in this systematic review. Most of these scales were developed using classical test theory (CTT) methods, which have inherent limitations [36], including imprecision in short questionnaires that might be suitable for large samples [37]. Additionally, the difficulty arises in comparing two similar questionnaires as each uses its own metrics [36].

One potential solution to these limitations is the adoption of computerized adaptive tests (CATs), constructed from a collection of items related to a specific subjective domain, as opposed to using the CTT. CATs, based on item response theory (IRT) banks, enable researchers and clinicians to capture the most informative items for each patient or group, providing a common metric independent of the choice of items [36]. Currently, specific CATs for anxiety are available [38,39].

Although the collected data provides valuable insights, a meta-analysis was not performed due to the significant heterogeneity among the included studies. This heterogeneity stemmed from differences in the types of anxiety disorders studied, variations in CBD dosages, and discrepancies in study designs. Such variability makes it challenging to aggregate the data into a cohesive statistical analysis without compromising the validity of the results. As a result, the decision was made to conduct a qualitative systematic review to better capture the nuances of the current evidence.

An important limitation of this study is the lack of registration in the PROSPERO database. Although not mandatory, PROSPERO registration could have enhanced the transparency and methodological rigor of this systematic review. This omission should be considered when interpreting the results, as it may affect the reproducibility and reliability of the findings.

This systematic review contributes novel insights by addressing a broader spectrum of anxiety disorders compared to previous meta-analyses. Unlike the studies cited by Fliegel and Lichenstein (2022) and Skelley et al. (2020), which focused on specific forms of anxiety or pooled data without differentiating between subtypes, our review evaluates a diverse array of anxiety disorders, offering a more comprehensive understanding of CBD’s efficacy. Furthermore, the inclusion of studies with varying dosages and methodologies highlights the nuances in treatment outcomes that were not fully captured in prior analyses. These contributions underscore the relevance of our findings for clinical practice, providing healthcare professionals with updated and detailed evidence to better guide treatment decisions involving CBD for anxiety disorders.

### Strengths and Limitations

The present study has some limitations that are worth mentioning. In our search strategy, we did not specify the type of anxiety because we wanted to observe the effects of CBD on anxiety in general. It is known within the scientific community that there are at least four types of anxiety, each with different symptoms: Generalized Anxiety Disorder (GAD), phobic disorders, panic disorders, and post-traumatic stress disorder (PTSD) [36].

The second limitation is that only one included study compared the effects of CBD with conventional pharmaceutical therapy on anxiety. Therefore, it is challenging to address the second part of the objective of this review.

The third limitation is that, even though the guidelines for quality appraisal were followed, many important items of the Cochrane Collaboration’s Tool were classified as unclear for risk of bias, consequently affecting the overall quality assessment of the studies included in this review.

## 5. Conclusions

Despite the conflicting results observed across the studies, our systematic review indicates that CBD shows promise in alleviating anxiety, particularly when compared to placebos, with a favorable safety profile and minimal adverse effects. The diversity of anxiety disorders evaluated and the range of CBD dosages analyzed in this review contribute to a more nuanced understanding of its therapeutic potential. However, the findings also underscore the need for more rigorously designed randomized controlled trials (RCTs) that address the limitations identified, such as the variability in study designs, inconsistent dosing regimens, and short-term treatment durations. Future studies should focus on standardized methodologies, including continuous CBD administration and long-term follow-ups, to better ascertain its efficacy across specific anxiety subtypes.

This review provides a critical update to the existing literature by expanding the scope of anxiety disorders studied and offering insights that previous meta-analyses may not have fully captured. By evaluating a broader spectrum of conditions and dosages, our study not only highlights the potential of CBD as an alternative treatment for anxiety but also lays the groundwork for future research aimed at optimizing its clinical use. Ultimately, these findings contribute to the ongoing discourse on integrating CBD into the therapeutic arsenal for anxiety disorders while also emphasizing the need for a continued investigation to solidify its role in clinical practice.

## Figures and Tables

**Table 2:** Summary of protocols, effects of CBD on anxiety, adverse effects of CBD, and study limitations.

Author(s) and Year of Publication	Groups and CBD Dose	Anxiety Measurement Methods	CBD Effects on Anxiety	CBD Side Effects	Study Limitations
Zuardi et al., 2017 [21]	- CBD 100 mg - CBD 300 mg - CBD 900 mg - CLON 1 mg - Placebo	- STAI - VAMS - Blood pressure - Heart rate	CBD 300 mg significantly relieved anxiety post public speech in comparison to placebo but did not reduce the blood pressure as much as CLON.	No side effects were mentioned	- small sample size; - did not have the results with other moderate doses of CBD (400 or 600 mg)
Hundal et al., 2018 [22]	- CBD 600 mg - Placebo	- BAI; - Blood pressure - Heart rate	No beneficial effects of CBD, and apparently, it increased the anxiety induced by paranoid thoughts.	CBD-treated group adverse events were tiredness/sedation, lightheaded/dizziness, nausea, abdominal discomfort, and increased appetite/hunger	- absence of plasma monitoring of CBD concentrations; - small sample size - single CBD dose
Linares et al., 2019 [16]	- CBD 150 mg - CBD 300 mg - CBD 600 mg - Placebo	- VAMS - Blood pressure	CBD 300 mg decreased anxiety during the speech in comparison with placebo.	No side effects were mentioned	- effects of CBD on subjective reports of anxiety and not physiological effects, - study was made only with men
Hurd et al., 2019 [23]	- CBD 400 mg - CBD 800 mg - Placebo	-VAS-A	CBD 400 mg and 800 mg reduced anxiety in heroin-abstinent volunteers.	No side effects were detected	- craving and anxiety outcomes were subjective self-reported measures, which may have provided unreliability or bias; - small sample size
Appiah-Kusi et al., 2020 [24]	- CBD 600 mg - Placebo	- STAI-S	CBD did not show any significant improvement on public speech—induced anxiety of people with high risk of psychosis.	No side effects were mentioned	- modest sample size; - stress caused by venepuncture to collect venous blood sampling; - they could not control other factors that could impact cortisol levels
Faria et al., 2020 [25]	- CBD 300 mg - Placebo	- VAMS - SSPS - Blood pressure - HR	CBD attenuated the anxiety induced by public speech on volunteers with Parkinson’s disease.	No side effects were detected	- small sample size; - time of CBD administration and the anxiety induction model should have been longer; - the anxiolytic effects observed cannot be generalized directly to any symptom of anxiety that may occur in daily living, since anxiety was induced experimentally in this study
Meneses-Gaya et al., 2021 [17]	- CBD 300 mg - Placebo	- BAI	CBD did not have any effect on anxiety induced by crack cocaine abstinence compared to placebo group.	- Sleepiness and increased sleep duration; nausea and headache	- symptoms tend to be less severe in hospitalized patients; - CBD dose is relatively low; - one single dose of CBD; - interference of other treatment outcomes once these patients were hospitalized more often; - sample may not represent the general socioclinical profile of crack cocaine users in general
Bloomfield et al., 2022 [26]	- CBD 600 mg - Placebo	- VAS-A - BAI - HR - Blood pressure	CBD did not have any effects on any measures relating to anxiety.	No side effects were mentioned	- the oral route of CBD administration was slow and associated with variable bioavailability; - relatively long fasting time and did not use an oil buffer, which may have led to insufficient absorption of CBD; - stimuli employed across tasks were inconsistent; - the mental arithmetic task was novel in the context of CBD research, whereas previous studies have employed public speaking
Hutten et al., 2022 [19]	- THC 13.75 mg - CBD 13.75 mg -THC/CBD 13.75 mg each - Placebo	- STAI—trait - VAS-A - EST	CBD, by itself, did not significantly change anxiety ratings on any of the anxiety measures induced by illicit drugs and alcohol abstinence. CBD relieved THC-induced anxiety completely when baseline anxiety was low, partly reduced THC-induced anxiety when baseline anxiety was medium and did not counteract THC-induced anxiety when baseline anxiety was high. CBD only counteracted THC-induced anxiety when trait anxiety was low.	No side effects were mentioned	- baseline state of anxiety was measured only by VAS and not by STAI and EST; -single dose of THC and CBD
Bolsoni et al., 2022 a [27]	- CBD 300 mg - Placebo	- IDATE - VAMS	CBD failed to attenuate increases in anxiety, alertness, and discomfort induced by the trauma recall; however, measures of cognitive impairment were significantly lower after recall in patients who received CBD compared to placebo.	No side effects were mentioned	- measurements were not subjected to correction for multiple comparisons; - presence of comorbidities as a variable in group matching was not included, which led to a greater concentration of participants with comorbidities in one of the groups (CBD) and hampered the interpretation of results.
Bolsoni et al., 2022 b [28]	- CBD 300 mg - Placebo	- VAMS - Blood pressure - Heart rate	In nonsexual trauma, CBD attenuated the increased anxiety and cognitive impairment of recall. However, it failed to do so when the event was sexual in nature.	No side effects were mentioned	- the mean patient age and time since the traumatic event were significantly lower among those with sexual trauma than nonsexual trauma, which could have influenced the nonattenuation of anxiety in the sexual trauma subsample; - the analysis was not corrected for multiple comparisons; - small sample size
Kwee et al., 2022 [18]	- CBD 300 mg - Placebo	- BAI - FQ - LSAS	CBD did not have any effect on anxiety induced by agoraphobia.	- Dizziness; drowsiness; tiredness; feeling of a strong blood flow	- suboptimal dose; - single dose of CBD, timing of administration and form of administration could impact the results
Mongeau-Pérusse et al., 2022 [20]	- Daily doses: CBD 800 mg - Placebo	- BAI - VAS-A - Levels of cortisol	No evidence for long-term administration of 800 mg CBD to be more efficacious than placebo for modulating anxiety symptoms and cortisol levels in individuals with cocaine use disorder.	No side effects were mentioned	- exclusion of individuals with other substances disorders limited the findings; - small sample size; - high attrition rate; - use of cocaine during the test may have masked the effects of cannabidiol during the weeks of its administration
Stanley et al., 2022 [29]	- CBD 150 mg - CBD 300 mg - CBD 600 mg - Placebo	- WTAS - VAS-A - VAMS - STAI-state - SSS-8	A single dose of CBD in different doses did not impact on anxiety triggered by test anxiety in college students, and a higher dose seemed to increase the anxiety symptoms.	No side effects were mentioned	- sample was not diverse; - CBD 300 mg showed anxiolytic effects on anxiety triggered by the speech test, but not on anxiety triggered by test anxiety; - test anxiety needed to be validated; - experimental manipulations were sensitive to inducing changes in TA specifically rather than anxiety symptoms broadly
Gournay et al., 2023 [30]	- Repeated doses: CBD 50 e 300 mg -Placebo	- DASS-A	Repeated 300 mg CBD administration for 2 weeks, but not an acute 300 mg dose, reduced anxiety symptoms compared to placebo.	- Somnolence; dry mouth; lightheadedness; nausea; headache; increased appetite	- differences in anxiety symptoms between groups at the baseline; - the current study exclusively relied on self-report indicators, leaving findings open to confounds related to affect and memory bias; - the modifications to the BMWS instructional set obfuscated the interpretation of acute effects

Note: CBD = cannabidiol; CLON = clonazepam; STAI = State–Trait Anxiety Inventory; VAMS = Visual Analog Mood Scale; BAI = Beck’s Anxiety Inventory; VAS-A = Visual Analog Scale for Anxiety; STAI-S = State–Trait Anxiety Inventory induced by stress; EST = Emotional Stroop Test; FQ = Fear Questionnaire; LSAS = Liebowitz Social Anxiety Scale; SPSS = Self-Statements during Public Speaking Scale; DASS-A = the anxiety subscale of the Depression, Anxiety, and Stress Scales-21, specific for anxiety; WTAS = Westside Test Anxiety Scale; SSS-8 = Somatic-Symptom Scale-8; IDATE = State–Trait Anxiety Inventory.

## Data Availability

No new data were created or analyzed in this study.
